# Mapping areas of spatial-temporal overlap from wildlife tracking data

**DOI:** 10.1186/s40462-015-0064-3

**Published:** 2015-11-01

**Authors:** Jed A. Long, Stephen L. Webb, Trisalyn A. Nelson, Kenneth L. Gee

**Affiliations:** School of Geography and Geosciences, University of St Andrews, Irvine Building, North Street, St Andrews, Fife, KY16 9AL UK; The Samuel Roberts Noble Foundation, 2510 Sam Noble Parkway, Ardmore, OK USA; Spatial Pattern Analysis & Research Laboratory, Department of Geography, University of Victoria, Victoria, BC Canada; Oaks and Prairies Joint Venture, Gene Autry, OK 73401 USA

## Abstract

**Background:**

The study of inter-individual interactions (often termed spatial-temporal interactions, or dynamic interactions) from remote tracking data has focused primarily on identifying the presence of such interactions. New datasets and methods offer opportunity to answer more nuanced questions, such as where on the landscape interactions occur. In this paper, we provide a new approach for mapping areas of spatial-temporal overlap in wildlife from remote tracking data. The method, termed the joint potential path area (jPPA) builds from the time-geographic movement model, originally proposed for studying human movement patterns.

**Results:**

The jPPA approach can be used to delineate sub-areas of the home range where inter-individual interaction was possible. Maps of jPPA regions can be integrated with existing geographic data to explore landscape conditions and habitat associated with spatial temporal-interactions in wildlife. We apply the jPPA approach to simulated biased correlated random walks to demonstrate the method under known conditions. The jPPA method is then applied to three dyads, consisting of fine resolution (15 minute sampling interval) GPS tracking data of white-tailed deer (*Odocoileus virginianus*) collected in Oklahoma, USA. Our results demonstrate the ability of the jPPA to identify and map jPPA sub-areas of the home range. We show how jPPA maps can be used to identify habitat differences (using percent tree canopy cover as a habitat indicator) between areas of spatial-temporal overlap and the overall home range in each of the three deer dyads.

**Conclusions:**

The value of the jPPA approach within current wildlife habitat analysis workflows is highlighted along with its simple and straightforward implementation and interpretation. Given the current emphasis on remote tracking in wildlife movement and habitat research, new approaches capable of leveraging both the spatial and temporal information content contained within these data are warranted. We make code (in the statistical software R) for implementing the jPPA approach openly available for other researchers.

**Electronic supplementary material:**

The online version of this article (doi:10.1186/s40462-015-0064-3) contains supplementary material, which is available to authorized users.

## Background

Through movement ecology, wildlife researchers continue to build a more detailed understanding of processes that shape wildlife movement patterns. The study of wildlife movement has been enhanced by advances in remote tracking (e.g., GPS, VHF, Argos) that continue to improve data quality, inference, and cost-effectiveness [1, 2]. Remote tracking offers unique opportunities for studying wildlife movement over broad spatial and temporal extents and at increasingly fine resolutions; these features help to address when, where, how, and why animals move [3]. While defining home ranges, the area used for normal wildlife activities [4], remains a common approach to exploring wildlife space-use patterns, increasingly high resolution tracking data makes analyzing more complex and detailed spatial-temporal patterns in wildlife movement and behaviour possible.

Inter- and intra-species interactions can play a key role in the movement patterns of many wildlife species. Behaviour arising from inter- and intra-species interactions can occur from a number of different wildlife movement processes, such as the development of social networks [5], mating [6], and territoriality [7]. For example, female white-tailed deer exhibit social behaviour in which they form matrilineal groupings for most of the year, except during parturition when females become solitary in preparation of, and immediately following, birth [8]. The aforementioned processes are examples of dependent behaviour in wildlife movement, where the movements of one individual influence another, or inter-individual interaction (commonly termed dynamic or spatial-temporal interaction [9, 10]). Observed patterns of social or interactive behaviour have important implications in the management of spatially explicit wildlife processes; one such process is disease spread, which is related to the spatial and temporal patterns of contacts between individual animals, either directly or indirectly [11, 12].

Exogenous factors (e.g., environment, landscape) are also known to shape the movement patterns observed in wildlife [3]. For example, wildlife movement processes and interactions are related to changing climatic and environmental conditions (e.g., range shifts, [13]), and increasing anthropogenic change (e.g., natural resource extraction, [14]). When the landscape changes, movement is often impacted and wildlife may be forced to shift their range and interact with other individuals or new species due to limited habitats and resource availability (e.g., water, [15]). Other landscape features can physically shape wildlife interactions by creating movement barriers and corridors [16–18]. In northern Alberta, Canada, Latham *et al.* [19] found that ungulates were utilizing anthropogenic cut-lines as movement corridors, which were in turn being used by predators to track prey, changing interacting patterns. The interplay between landscapes and wildlife interactions influence health and survival, and due to the emphasis on wildlife management through landscapes, have substantial implications [20, 21].

Studies examining interactions using wildlife tracking data typically aim to quantify one of two conceptually related yet unique joint movement processes: 1) spatial-only interaction, and 2) inter-individual interaction [9, 10]. Methods for exploring spatial-only interaction (often termed static interaction, [9]) involve quantifying the joint space use between two individuals, often through calculation of the overlap zone (OZ) of two home ranges. Extending the home range OZ is the volume of intersection measure, which delineates the joint probability of occurrence of two animals whose movements are characterized by two different utilization distributions [22]. However, neither the OZ nor the volume of intersection incorporates timing of joint space use, or likelihood of wildlife encounters. While spatial-only interaction measures highlight areas utilized by multiple wildlife [23]; high temporal resolution tracking data enables the possibility of quantifying the likelihood of wildlife encounters [24, 25].

Inter-individual interactions can be analyzed using a suite of indices that test for the presence of interactions in tracking data (reviewed by [25]). These methods define inter-individual interaction associated with *contacts* measured using spatial (*d*_*c*_) and temporal (*t*_*c*_) thresholds to identify when location fixes co-occur in space and time. Contact based measures of inter-individual interaction test the observed number of contacts against an expectation or null model in order to identify attraction (higher than expected contacts) or avoidance (lower than expected contacts) behaviour [26, 27]. Other methods exist to study altogether different aspects of inter-individual interaction patterns in wildlife tracking data such as coordinated movement [28, 29], group-dynamics [30], and flocking or herding [31, 32].

A major limitation of currently available indices of inter-individual interaction is that they do not facilitate a spatially explicit measure of *where* interactive behaviour occurs on the landscape. Wildlife are known to select areas within their home ranges unevenly [33] and we expect interactive behaviour to also exhibit different spatial patterns. In this paper, we propose a new method for mapping areas of spatial-temporal overlap from wildlife tracking data. Mapping areas of spatial-temporal overlap will provide new avenues for research aimed at studying the linkages between interactive behaviour and environmental factors. The new approach draws upon previous work using time geography to estimate wildlife home ranges [34], and extends methods used to study interactions in human movement studies [35] to the study of wildlife movement. First, we introduce the theory of time geography and its current application to wildlife studies and then describe the potential path area (PPA) approach to delineating the home range and how we extend this to compute a new measure of spatial-temporal overlap. Following this, we demonstrate the new method with simulated and empirical data. We finish with discussion of our findings and future opportunities for studying inter-individual interactions from wildlife tracking data.

## Methods

### Background – time geography

**Fig. 1:**
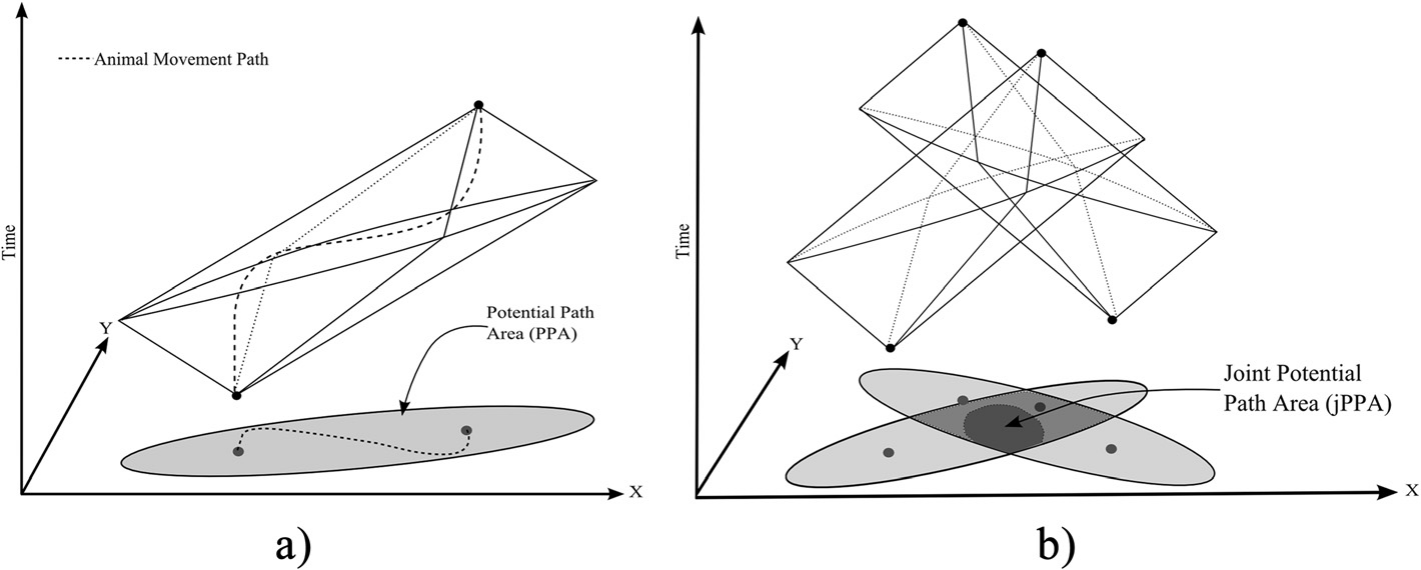
**a** Space-time prism, between two known fixes, projected onto the geographical plane and associated potential path area (PPA). **b** Intersection of two space-time prisms, and the projection of the joint accessible space onto the geographical plane – the joint potential path area (jPPA). In (**b**) light grey represents individual home ranges, medium grey represents home range overlap zone, and dark grey the jPPA; a spatial measure of inter-individual interaction

Time geography [36] represents a powerful framework for exploring how different spatial-temporal processes influence individual movement. The space-time prism (Fig. 1a) represents the conceptual building block for time geographic analysis and delineates the potentially accessible locations in space and time for an individual, conditioned on known start and end positions and a measure of mobility; with wildlife tracking data, *n*-1 space-time prisms can be constructed from a dataset comprised of *n* fixes. The space-time prism can be projected onto the geographic plane in order to map the potential path area (PPA), which is a polygon representing accessible areas to movement. The mathematical definitions for time geography are rigorously laid out by [37].

The space-time prisms of two individuals can be intersected in order compute the joint accessibility space – termed the social interaction space [35]. The social interaction space represents a measure of the areas, in space and time, where direct inter-individual interaction is possible (i.e., contact between the two individuals are only possible within the spatial-temporal boundaries of the social interaction space). The social interaction space can be projected onto geographical space in order to create a map of spatial-temporal overlap – termed the joint potential path area (jPPA – Fig. 1b). Note that the jPPA is fundamentally different from a measure of home range, or spatial overlap, as it explicitly shows only those regions where two individuals have the potential to ‘meet’ in space and time.

### Calculating the PPA

**Fig. 2:**
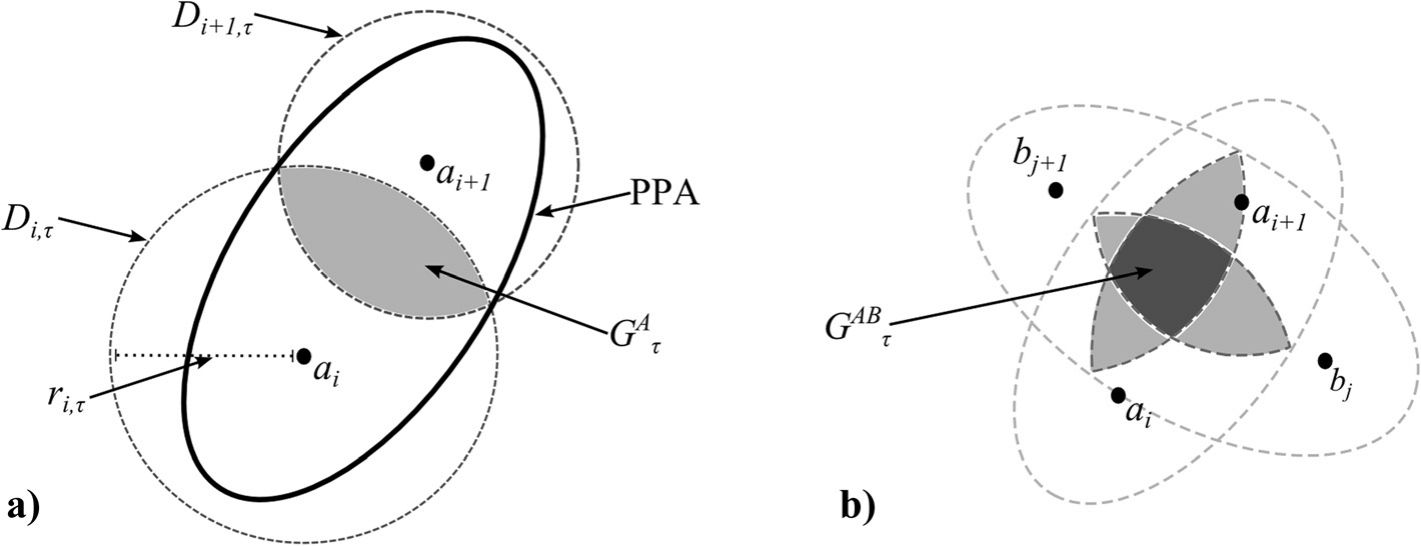
**a** The accessibility space (*G*_*τ*_) of an animal at time *τ* (*i* < *τ < i* + 1), where movement begins at known fix location *a*_*i*_ and ends at known fix location *a*_*i+1*_, is the intersection of the forward disc (*D*_*i,τ*_) and past disc (*D*_*i+1,τ*_). The union of the *G*_*τ*_, for all *τ*, is then the PPA; which by definition is an ellipse. **b** The joint accessibility space (*G* ^*AB*^_*τ*_) of two animals (*A* and *B*) at time *τ* (*i*, *j* < *τ < i* + 1, *j* + 1),, where movement begins at known fixes *a*_*i*_, *b*_*j*_, and ends at known fixes *a*_*i+1*_, b_j+1_ respectively, is the intersection of *G* ^*A*^_*τ*_ and *G* ^*B*^_*τ*_

Consider tracking data of an individual animal (A) corresponding to a set of *n* location fixes collected at discrete times A = {*a*_*1*_, *a*_*2*_, *a*_*3*_, … *a*_*n*_}, where *a*_*i*_ represents the location fix of the individual at time *t*_*i*_. Thus, for any point in time *τ* let {*a*_*i*_, *a*_*i+1*_} be two sequential fixes such that *t*_*i*_ < *τ* < *t*_*i+1*_ (Fig. 2a). Following [37], let *D*_*i,τ*_ be a disc centered on the first point (*a*_*i*_) with radius (*r*_*i,τ*_) defined by:

1 $$ {r}_{i,\tau }={v}_{\max}\times \left(\tau -{t}_i\right) $$

Where *v*_*max*_ is a parameter related to animal mobility (i.e., a maximum travelling velocity), and (*τ* - *t*_*i*_) is the time difference between *τ* and *t*_*i*_. Similarly, let *D*_*i+1,τ*_ be a disc centered on the second point (*a*_*i+1*_), with a radius (*r*_*i+1,τ*_) defined by:

2 $$ {r}_{i+1,\tau }={v}_{\max}\times \left({t}_{i+1}-\tau \right) $$

The intersection of *D*_*i,τ*_ and *D*_*i+1,τ*_ represents the accessibility space for individual A at time *τ* (termed *G*_*τ*_ – Fig. 2a).

3 $$ {G}_{\tau }={D}_{f,\tau}\cap {D}_{p,\tau } $$

For any pair of sequential fixes (*a*_*i*_, *a*_*i+1*_), the PPA is defined as the union of all *G*_*τ*_, such that *τ* is on the interval [*t*_*i*_, *t*_*i+1*_] [37].

4 $$ PPA={\displaystyle \underset{\tau ={t}_i}{\overset{t_{i+1}}{\cup }}{G}_{\tau }} $$

In application, calculation of the PPA is straightforward because the PPA is a perfect ellipse, the parameters of which can be calculated directly from the fixes and the mobility parameter *v*_*max*_ (see [34]).

### Calculating the jPPA

In order to quantify spatial-temporal overlap in wildlife tracking data, we delineate when and where the space-time prisms of two individual animals intersect, by calculating overlap in the accessibility spaces (*G*_*τ*_) of two individuals (A, B), which we denote *G*_*τ*_^*A*,*B*^ (Fig. 2b).

5 $$ {G}_{\tau}^{A,B}={G}_{\tau}^A\cap {G}_{\tau}^B $$

The joint potential path area (jPPA) is then defined as the spatial union of the *G*_*τ*_^*A*,*B*^ for each *τ* that is within the interval of [*t*_*i*_, *t*_*i+1*_] (Fig. 2b).

6 $$ jPPA={\displaystyle \underset{\tau ={t}_i}{\overset{t_{i+1}}{\cup }}{G}_{\tau}^{A,B}} $$

By definition, the jPPA will always be a sub-region of the intersection of the two PPA ellipses (see Fig. 1b) because the intersection of two ellipses generated by the PPA is the measure of the spatial-only overlap, while the jPPA represents spatial-temporal overlap. In order to compute the jPPA for a larger tracking dataset, we must recursively compute *G*_*τ*_^*A*,*B*^ for all overlapping τ within the temporal period when animals A and B were simultaneously tracked.

### Simulation study

A simulation study was used to examine the jPPA method and contrast it with the commonly employed spatial overlap measure in order to evaluate the ability of each for identifying and mapping interactive movement behaviour. Correlated random walks (CRW – [38, 39]) and biased correlated random walks (BCRW – [40, 41]) were used to generate simulated tracking data where inter-individual interactions would be expected and unexpected (null case). Four scenarios were implemented representing different types of inter-individual interactions commonly encountered in wildlife systems: i) no interaction, ii) grouping, iii) leading/following, and iv) joint resource use. In the first scenario, no interaction is simulated via two independent CRW, the second originating within the minimum convex polygon of the first. In the second scenario, grouping, a CRW is used to simulate the dynamic location of a group centroid, and the two individuals movements are biased towards this location [30]. In the third scenario, leading, the movements of the second individual are biased towards the current position of the first [25]. In the fourth scenario, joint resource use, the movements of the two individuals are biased towards a patch collocated between two home ranges [40]. We allowed the simulated animals to switch between CRW and BCRW with some fixed probability at each step, to emulate a behavioural switching between interactive and non-interactive phases [42]. Simulations where the spatial overlap in home ranges was < 10 % of the combined home range area were discarded corresponding to the idea that spatial overlap is a pre-cursor for spatial-temporal interaction [25]. Similarly, in the scenarios 2, 3, and 4 we discarded all simulations where < 10 % of the time was spent in the interactive phase to ensure these scenarios demonstrated the expectation of interaction. We ran the simulation process for each scenario until 1000 simulations were achieved. More details on the simulation procedure, and accompanying R code, can be obtained from the Additional file 1.

For each simulated animal, we first computed the PPA estimate of home range [34]. We then computed a measure of spatial-only interaction, the overlap zone defined as OZ = A∩B, where A and B are the PPA home ranges of the two simulated individuals. Then we computed the jPPA. To facilitate straightforward comparisons, the areas of the OZ and jPPA were normalized by the total joint home range area (A⋃B), such that each measure ranged from 0 to 1; 0 indicating no spatial overlap, and 1 complete spatial agreement. Evaluating the simulations was done by considering the 1000 runs from the first scenario (no interaction) as a test distribution representing where interaction is unexpected. Then we would expect that the values of the OZ and jPPA in the three scenarios where interaction was expected to lie in the outer tails of this distribution. In such a randomization test, the one-sided test statistic is computed as the probability *p* = (*n*_*e*_ + 1)/*n* of getting a value equal to or more extreme in the null distribution in comparison to the observed value, where *n*_*e*_ is the count of these extreme values. Using a critical value of *α* = 0.05, we examine the ability of the jPPA against the naive OZ statistic for identifying expected interaction behaviour. We contrast the performance jPPA against the OZ statistic in comparison to the OZ. Further, for the three simulated scenarios where BCRW were used to simulate known interactive behavior, we further contrast the jPPA with the proportion of time where the two animals were in the BCRW interactive phase (termed pInt). The value for pInt can be considered an indicator of the known or true level of interaction in BCRW simulations.

### Empirical data: GPS tracking of white-tailed deer

We captured 38 white-tailed deer (*Odocoileus virginianus*) from a study area in south-central Oklahoma, USA from 1998 to 2004. The study site was 1,214 ha, and was surrounded by a 15-strand, high-tensile electric fence (2.5 m tall), thus partially restricting movement across property boundaries [43]. Vegetation was consistent with that of the Cross Timbers and Prairies ecoregion [44]. Deer were captured during the winter months using modified drop-net systems [45] and fitted with GPS collars (ATS G2000 remote-release collars; Advanced Telemetry Systems, Inc., Isanti, MN) programmed to collect fixes at a 15 min sampling interval. Data were successfully retrieved from 32 of 38 GPS collars. All capture, handling, and marking procedures were consistent with the guidelines of the American Society of Mammalogists [46] and were approved by permit from the Oklahoma Department of Wildlife Conservation.

We performed jPPA analysis on six deer representing three unique deer dyads (Table 1) in order to demonstrate the jPPA approach with empirical GPS tracking data. For dyad 1, two males during and after mating season, we predict little inter-individual interaction between the two individuals. With dyad 2, one male and female during rut, we predict a much different pattern where a period of sustained inter-individual interaction may be an indication of courtship and mating behaviour. Last, for dyad 3, two males during late winter, we predict greater inter-individual interaction as a result of the formation of bachelor groups. With empirical data, we generally do not have a known level of interaction, thus we use a simple statistic, the proportion of simultaneous fixes that are spatially proximal (within a distance threshold of 50 m) to estimate the level of interaction between individuals.

**Table 1:** Sex, age, and the tracking period of six white-tailed deer that were part of the empirical analysis of jPPA; the 6 deer represented 3 unique dyads

Dyad	Deer ID	Sex	Age	Tracking Period
1	24	M	3.4	21-Nov - 08-Feb
	25	M	4.4	21-Nov - 06-Dec
2	34	M	6.4	23-Nov - 17-Jan
	35	F	4.5	14-Dec - 17-Feb
3	13	M	2.7	15-Feb - 28-Mar
	14	M	2.7	15-Feb - 21-Mar

For each individual deer, we computed the PPA estimate of individual home range [34]. From the PPA home range estimates, we computed the spatial overlap zone (OZ), similar to previous home range overlap analysis [23, 47] as a measure of spatial-only interaction. Finally, we computed the jPPA for each dyad. Calculating the PPA and jPPA home range areas requires estimating the *v*_*max*_ parameter, which represents an upper bound on mobility. We expect deer to show higher levels of mobility at dawn and dusk than during the night and day; therefore, we estimated the *v*_*max*_ parameter for the PPA and jPPA dynamically [48] for four time periods throughout the day: dawn (05:00 – 09:00), day (09:00 – 17:00), dusk (17:00 – 19:00), and night (19:00 – 05:00) following temporal intervals from [49]. In all cases, we used the van der Watt [50] method (with *k* = 5) for estimating *v*_*max*_ from the tracking data following [34, 48].

We investigated differences in vegetation within each of the individual home ranges, the OZ, and the jPPA for each of the three deer dyads. We chose a single metric – percent canopy cover – as a representative indicator of habitat. Vegetative cover is an important habitat component for white-tailed deer because it helps regulate the local thermal environment and provides concealment against predators [51, 52]. Percent canopy cover data were obtained from the US National Land Cover Database (NLCD, [53]). The NLCD percent canopy cover data are derived from Landsat satellite imagery and are represented at a spatial resolution of 30 m. For each of the three deer dyads, we calculated the mean and standard deviation of percent canopy cover (i.e., the mean of all pixels) for the pixels associated with the original telemetry points, the area within each individual’s PPA home range, the OZ, and the jPPA. Further, we examined the distribution of the percent canopy cover values associated with the individual points and within each of the home range and joint areas using overlaid density plots (with a bandwidth of 10) to explore variability in use of canopy cover across the range of potential values (0 – 100 %).

## Results

### Simulation study

**Fig. 3:**
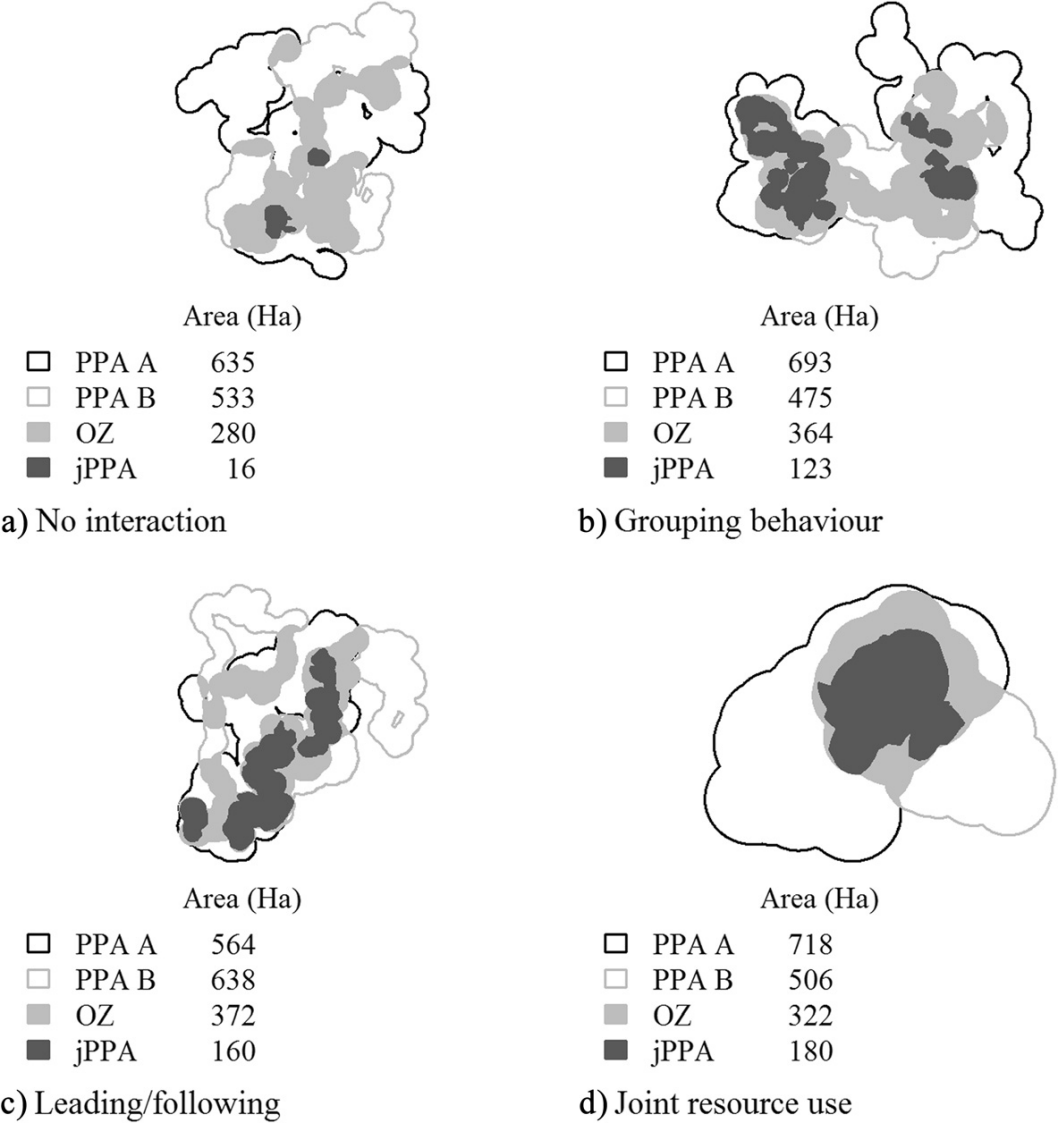
Simulated dyads from each of the four scenarios, to exemplify the different types of interaction simulated using correlated and biased correlated random walks: **a** no interaction, **b** grouping, and **c** leading/following, **d** joint resource use. Figures show the potential path area (PPA) estimate of home range, the spatial overlap zone (OZ), and joint potential path area (jPPA)

It is not practical to view the map of spatial-temporal overlap associated with each of the 1000 simulations for each of the four scenarios, but we provide an example of each scenario for illustrative purposes (Fig. 3). In scenario 1, where no interaction is expected, some spatial-temporal overlap is still possible, due to random or chance encounters (Fig. 3a). Scenario 1 serves as the basis for the null distribution, which we use to test against the other three distributions. In the second scenario, grouping behaviour may occur during different phases resulting in different locations of spatial-temporal overlap. Similarly, in scenario 3, individuals may exhibit consistent periods of spatial-temporal overlap resulting in disjoint jPPA patches (Fig. 3c). Finally, in scenario 4, a jointly utilized resource patch serves as the focal location for a single area of spatial-temporal overlap (Fig. 3d).

**Fig. 4:**
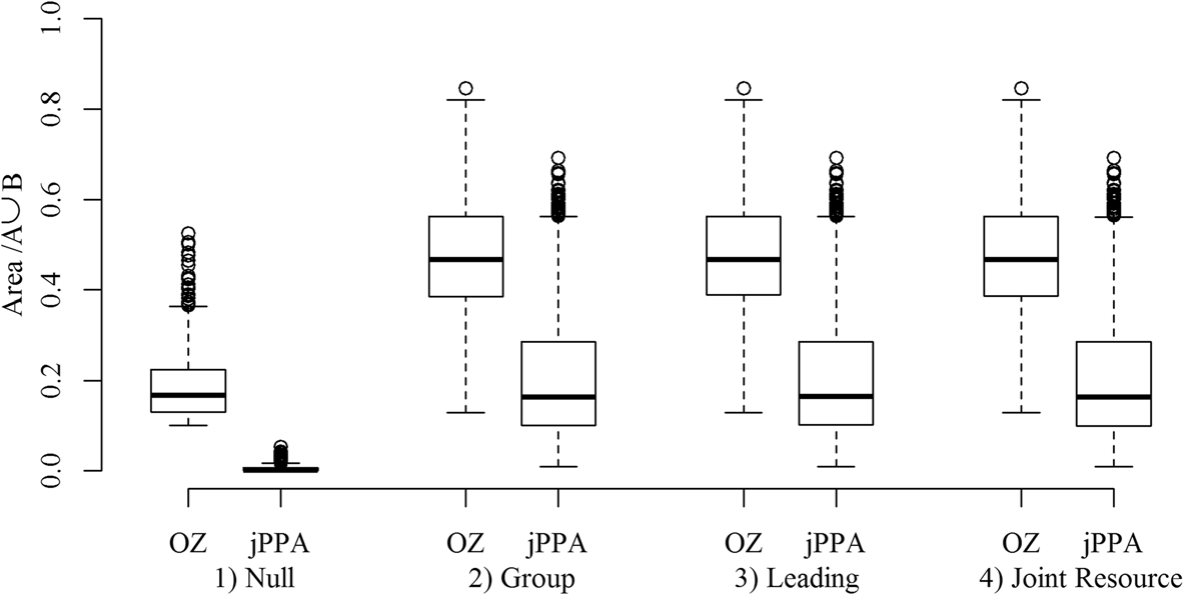
Boxplots showing the distribution of the proportional area of the overlap zone (OZ) and joint potential path area (jPPA), normalized by the area of the union of the two deer potential path area (PPA) home ranges for each simulation scenario (1000 simulations were run for each scenario)

It is first useful to examine the distribution of results associated with each of the scenarios (Fig. 4). From the boxplot in Fig. 4, we can see two general differences between the areas of the OZ and the jPPA. The first difference is that on average, the OZ is much larger than the jPPA in all scenarios. For example, for scenario 2: grouping behavior, the median value of the proportional area of the OZ is 0.47 while the median of the proportional area of the jPPA is 0.16 (Fig. 4). The second, and perhaps most important, distinction is the ability of the jPPA to correctly identify the absence of interaction in the first scenario containing independent CRW, which we use as a null distribution for significance testing. Here, the median value of the proportional area of the OZ is 0.17 with an interquartile range of 0.09, while the median value of the proportional area of the jPPA is 0.002 with an interquartile range of 0.007.

**Fig. 5:**
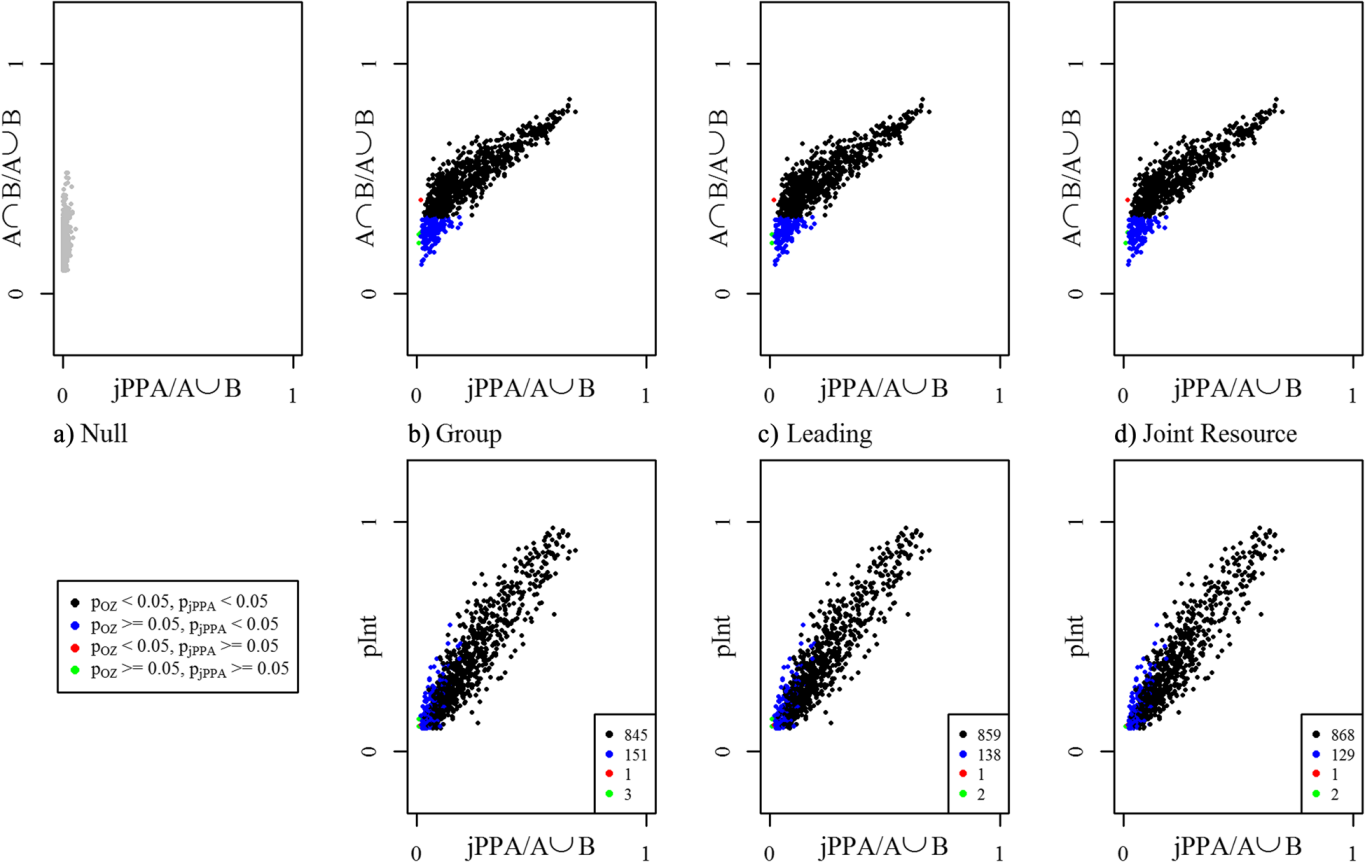
Results from the simulation study where pairs of animals were simulated using two independent correlated random walks (**a**) no interaction expected, and biased correlated random walks defined as (**b**) group behaviour, (**c**) leading behaviour, (**d**) joint resource use, where interaction is expected. The scatter plots show the number of simulations where *p* < 0.05 for each of the three interaction scenarios using the case of no interaction as the null distribution. In the top row, the area of the jPPA divided by the total area of the union of the two home ranges is plotted on the x-axis against the area of the OZ divided by the union of the two home ranges on the y-axis. In the bottom row, the area of the jPPA divided by the total area of the union of the two home ranges is plotted on the x-axis against the proportion of time each simulation spent in the interative phase (pInt) on the y-axis. The pInt value is always zero for the Null scenario, and thus this plot is not included. Black points indicate where both the jPPA and naïve OZ measure correctly identify interaction, while blue indicates where the OZ measure did not identify interaction. Red points indicate where jPPA did not identify interaction, but the OZ measure did, while green points indicate where both methods did not identify interaction

With scenario 2, grouping, the jPPA identified 996 of 1000 simulations as having interaction when compared to the null distribution (*p* < 0.05), while the OZ identified 846, only 3 cases were deemed as having no interaction by both measures (Table 2). In scenario 3, leading, the jPPA identified 997 of 1000 simulations as having interaction, while the OZ identified only 860, and only 2 cases were deemed as having no interaction by both measures (Table 2). Finally, in scenario 4, joint resource use, the jPPA identified 997 of 1000 simulations as having interaction, while the OZ identified only 871 (Table 2). Further inspection of the null distribution (Fig. 5a) demonstrates the issue with using the OZ as a measure of direct interaction and inter-individual movement behaviour, as relatively high OZ values (e.g., OZ > 0.4) can easily occur when no interaction is present. We also see similar results in each of the three scenarios where interaction was simulated via BCRW. If we use the proportion of fixes in each simulation spent in a biased phase (pInt) (bottom row, Fig. 5) as a measure of the true level of interaction, we can see that the OZ measure often fails to correctly identify cases (blue points) with substantial levels of interaction (e.g., pInt > 0.3). The few cases where the jPPA fails to identify interaction in these simulations, are cases where pInt was relatively low. Our results suggest that at the very least the jPPA is much more suitable than the OZ for characterizing different types of inter-individual movement. Thus, maps of jPPA polygons represent a suitable starting point for exploring landscape covariates associated with inter-individual movement behavior measured as spatial-temporal overlap.

**Table 2:** Performance of the OZ and jPPA method at correctly identifying the presence of interaction from the three simulation scenarios: group behavior, leading/following, and joint resource use (1000 simulations were run for each scenario)

		Sig.^a^ interaction	Sig.^a^ interaction identified by only one method	No sig.^a^ interaction identified
Scenario 2:	OZ	846	1	3
Group behaviour	jPPA	996	151	
Scenario 3:	OZ	860	1	2
Leading/following	jPPA	997	138	
Scenario 4:	OZ	869	1	2
Joint resource use	jPPA	997	129	

^a^Significant interaction defined using null distribution of 1000 independent correlated random walk (CRW) pairs and a critical value of *α* = 0.05

### Empirical data: GPS tracking of white-tailed deer

**Fig. 6:**
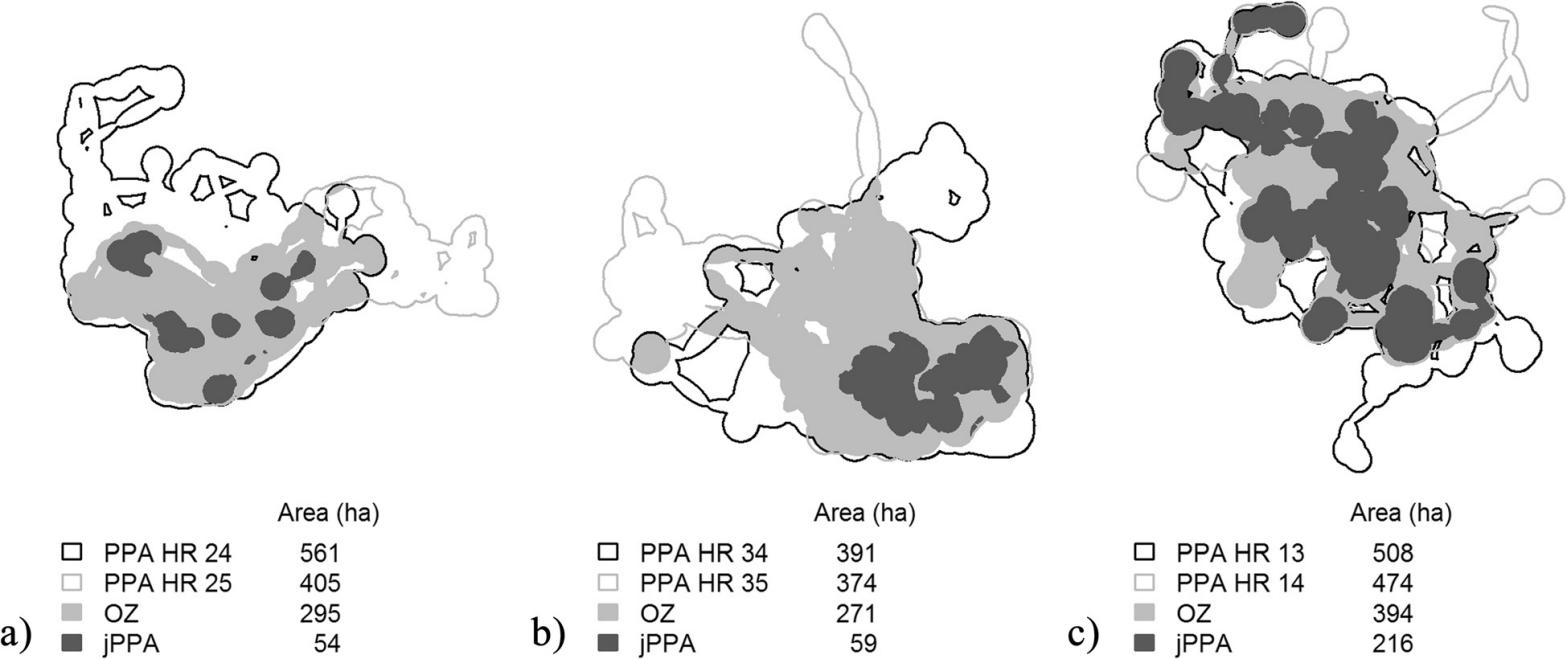
Three white-tailed deer dyads in Oklahoma, USA, **a** dyad 1, two adult males; **b** dyad 2, an adult male and female; and **c** dyad 3, two adult males. PPA estimates of individual home ranges, along with the overlap zone (OZ), and the joint potential path area (jPPA) are shown. The areas (in ha) of each spatial unit are provided alongside the legend

The three deer dyads reveal different patterns of space use and inter-individual interaction as evident by the maps in Fig. 6. In the first dyad, there is a relatively large area where the two home ranges overlap (OZ), and within the OZ there are dispersed, but small, spatial regions that jPPA identified as potential inter-individual interaction between the two deer (Fig. 6a). Further analysis corroborates the finding of little inter-individual interaction, which could be described as random interactive encounters as evidenced by a low proportion of proximal simultaneous fixes (1.9 % of simultaneous fixes within a critical distance of 50 m). In the second dyad, there is a greater area of overlap between the two deer’s home ranges (OZ), with the jPPA representing only a small proportion (21.8 %) of the OZ area. However, the jPPA is spatially more contiguous (Fig. 6b). Again with the second dyad, we find that only a small proportion of simultaneous fixes within the critical distance of 50 m (2.6 %). The third dyad shows a more substantive level of the jPPA observed throughout the PPA ranges and overlap zone of the two individuals (Fig. 6c). The jPPA comprised 54.8 % of the OZ stemming from the fact that 18.7 % of simultaneous fixes were within the critical distance of 50 m.

**Fig. 7:**
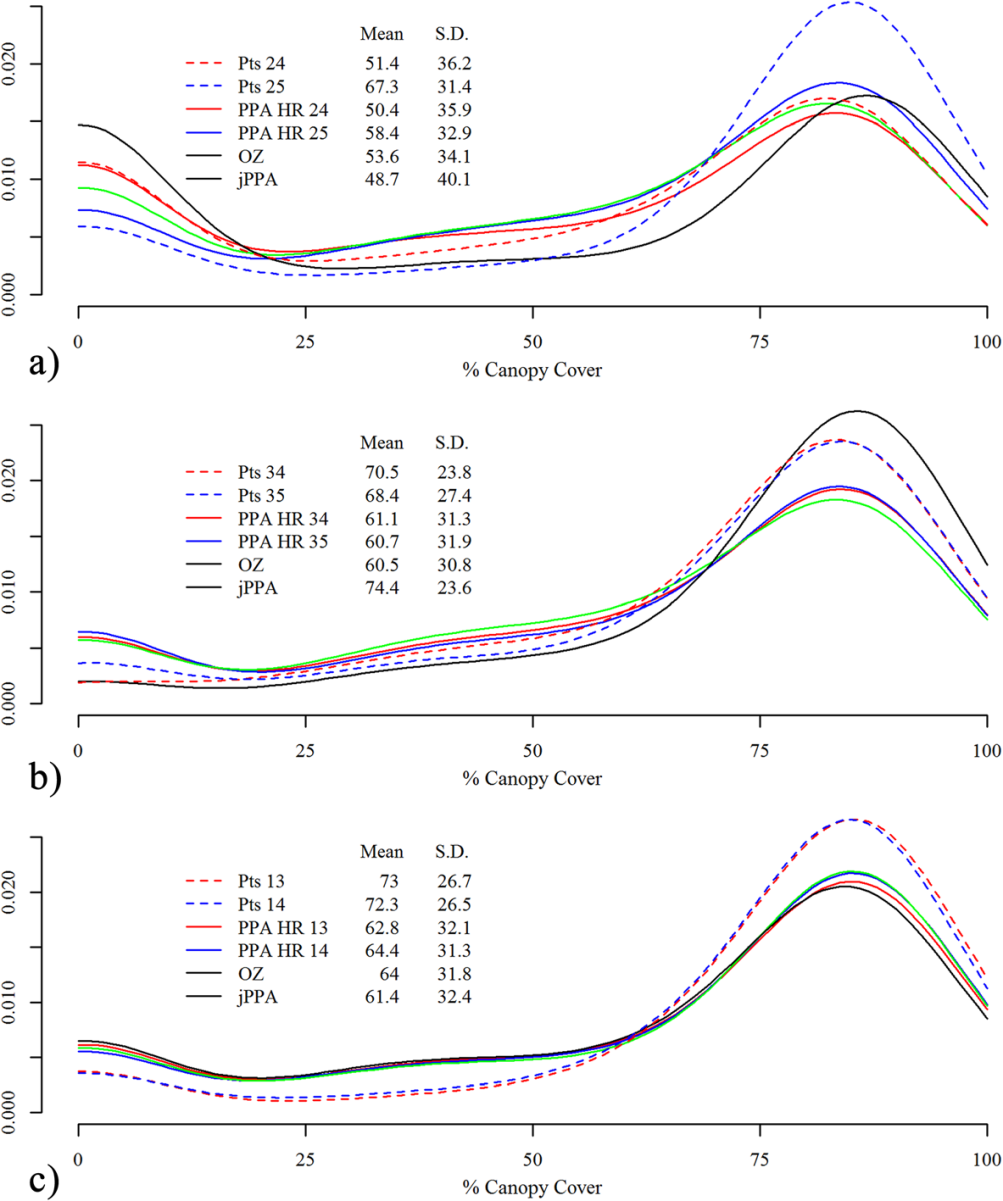
Density plots showing the relative proportion of percent canopy cover pixel values associated with the original telemetry points (Pts) of each individual, within each of the individual potential path area (PPA) home ranges, the overlap zone (OZ) and the joint potential path area (jPPA) measure of spatial-temporal overlap; **a** deer dyad 1, two adult males after the rut; **b** deer dyad 2, an adult male and female; and **c** two adult males during late winter

To show broader application of the jPPA method, we analyzed percent canopy cover by white-tailed deer associated with the original telemetry points, and across the three spatial areas (PPA home range, OZ, and jPPA). The two male deer in dyad 1 showed slightly different use of canopy cover associated with their telemetry points (51.4 vs. 67.3 %) and within their respective home ranges (50.4 vs. 58.4 %; Fig. 7a). Canopy cover within the OZ fell between the two estimates for the individual home ranges, with canopy cover lower in the jPPA (48.7 %) compared with the other areas. The density plots for each of these regions suggests selection for use of both forested and open areas, with deer 25 showing stronger preference for forested areas. Areas where interaction occurs (as defined by the jPPA) appear with higher probability in open regions (Fig. 7a). In the second dyad, one adult male and one adult female, we observed that canopy cover was similar across the individualhome range areas and the OZ (~61 % Fig. 7b); but higher as defined by the raw telemetry points (68-71 %). Canopy cover in the jPPA (74.4 %) was found to be greater than defined by home ranges, OZ, and telemetry points. The density plots reaffirm the preference for forested areas during interactive stages, with the density plot for the jPPA lying well above those for the home ranges and OZ at higher percent canopy cover levels, and somewhat higher than those for the raw telemetry points. In the third dyad of two male deer, the jPPA, overlap zone, and PPA home ranges had similar levels of canopy cover as indicated by both the mean values, and the density plot curves, which were very similar in shape (Fig. 7c). The curves associated with the raw points suggest a stronger selection for forested area over open areas, but a similar pattern to that of the home ranges, OZ, and jPPA.

## Discussion

### The time geography movement model

The time geographic approach to animal space-use analysis is aided by its simple implementation and interpretation. Unlike current methods for studying inter-individual interaction, the jPPA method focuses on mapping inter-individual interactions, defined as the area of spatial-temporal overlap. Output polygons from the jPPA are easily interpreted and can be readily integrated into existing workflows common to many wildlife movement studies (e.g., home range and habitat analysis). The jPPA method does not facilitate a statistical test for the presence of significant inter-individual interaction, thus, the jPPA compliments the existing suite of methods for studying spatial and inter-individual interactions currently available to wildlife researchers.

Conceptually, the jPPA extends the time-geographic approach for quantifying space use in wildlife tracking data [34, 54], which explicitly considers the temporal ordering of fixes and a parameter of mobility in order to quantify what is termed the *accessibility space* of an individual. The jPPA is then defined as the *joint accessibility space* of two individuals, and has been successfully applied to numerous studies of human mobility and transportation patterns [35, 55]. The jPPA should therefore be interpreted as the spatial region delineating the areas that are jointly accessible, in space and time, by two animals. Herein, we introduced the jPPA with *k* = 2 individuals (termed dyads), but the same calculation can be extended to *k* > 2 individuals to delineate the joint accessibility space of a larger group of animals. For example, local resources may be simultaneously utilized between multiple species or wildlife assemblages. Where tracking data of multiple individuals are recorded simultaneously, the jPPA method can be used to locate these areas across the landscape.

Space-time prisms (which are the basis for the jPPA) are straightforward to construct from tracking data (e.g., fix locations and times), along with a parameter describing the mobility of the animal (termed *v*_*max*_), which is interpreted as maximum travelling velocity. It is best to consider *v*_*max*_ as a function of the sampling interval; for example, *v*_*max*_ will change depending on whether tracking data were collected with a 10 min or 2 h sampling interval. The estimation of *v*_*max*_ can be based on expert knowledge of animal biology or using statistical procedures based on the tracking data [34]. When estimating *v*_*max*_ from the tracking data it is useful to consider the distribution of the observed segment velocities (*v*_*i*_) given by *v*_*i*_ = *d*_*i*_ / *t*_*i*_, where *d*_*i*_ is the distance and *t*_*i*_ the time between consecutive fixes. Based on the full distribution of the *v*_*i*_, statistical estimation procedures used for estimating the upper-bound of a distribution [50, 56] can be applied to estimate *v*_*max*_. Mobility levels change over space and time, thus the *v*_*max*_ parameter can be modeled dynamically to better reflect changes in individual behaviour or travel mode [48].

The jPPA is most appropriately applied where tracking data are collected with a moderately high resolution and regular fix interval (i.e., such as the white-tailed deer data here using a 15 min interval). This is because the jPPA method explicitly considers the sequential ordering and the time duration between fixes in its derivation (i.e., equations (1), (2)). When the sampling interval is extremely high (e.g., some species are now being tracked at ≤ 1 min intervals, [57]) the potential for movement between known fixes is limited, and thus the PPA will be small. Similarly, when the sampling interval is extremely coarse, the potential for movement between known fixes is substantial, and thus the PPA will be large. In the first case, the jPPA is likely to underestimate potential interaction because of a mis-match between the data resolution and the functional scale of interaction (e.g., defined by sight, smell, sound etc.). In the second case, the jPPA will over-estimate interaction because of the immense possibility of potential movement when fixes are taken infrequently (e.g., daily or less). Much like the PPA estimate of home range [34, 48], jPPA should be used with caution with much coarser sampling resolutions because of the uncertainty associated with the delineation of the potential path area when the time duration between fixes is long. However, with high-resolution tracking data, the jPPA may be computed at multiple resolutions via re-sampling of the original tracking data. Plotting jPPA results against different tracking resolutions may provide insights into the functional scale of movement interaction, considering both the mobility of the animal and the potential spatial range associated with interactions. For example, questions may begin by discovering at what sampling interval the jPPA becomes significant, and then begin to consider what movement processes might correspond to this interval (e.g., fine vs. coarse scale movement).

### Studying inter-individual interactions from wildlife tracking data

The jPPA provides an alternative approach to studying inter-individual interactions from wildlife tracking data, one that focuses specifically on spatially explicit mapping of inter-individual interaction. Unlike previously developed indices of inter-individual interaction, the jPPA does not facilitate a formal statistical test of the presence (or absence) of inter-individual interaction. Statistical tests for inter-individual interaction from wildlife tracking data can be problematic, owing to the issue of generating appropriate null distributions from which to test against [26, 27, 58]. Rather, the jPPA is able to detect, and more importantly map, infrequent and/or random inter-individual interaction areas (e.g., chance encounters) across the landscape. Such infrequent or random interactions typically go undetected when using formal statistical tests because they do not constitute a statistically significant interaction [25]. Because of their importance in shaping biological processes, such as the spread of disease and predator–prey dynamics, methods capable of identifying and mapping random or unexpected encounters, such as jPPA, offer new potential for studying infrequent or random interactions by wildlife using tracking data.

The jPPA approach can be compared to existing measures of static interaction; defined as the spatial area used jointly by two (or more) animals [9, 10]. We compared the jPPA to the most common measure of static interaction, the overlap zone, defined as the spatial intersection of two home ranges. When the PPA home range estimate is employed, the jPPA will be a spatial sub-region of the OZ and represents those areas where animals could have potentially ‘meet’ in both space and time. Recent research has suggested that the utilization distribution, which represent space use as an uneven probability surface [59, 60], may be a more useful spatial measurement as animals typically use different areas of their home ranges unevenly in space and time [61]. The volume of intersection [22, 33] then represents the analogous spatial interaction measure for utilization distributions; delineating a map showing the joint space-use probabilities. However, the volume of intersection of two (or more) utilization distributions does not explicitly consider time (i.e., simultaneous joint space use). Thus, the jPPA can be considered the spatial-temporal extension of the home range overlap zone and represents a discrete map (i.e., a polygon) of where inter-individual interaction was possible. Further extending the jPPA using some probabilistic models [62, 63] could facilitate probabilistic statements similar to those from the volume of intersection measure for studying inter-individual interaction probabilities.

New types of tracking sensors (i.e., proximity loggers) are capable of directly estimating inter-individual interactions (i.e., contacts) by sensing when, and for how long, two tagged animals are within a defined distance threshold [64]. However, proximity loggers are not designed to record where contacts occur, and the actual distance between two animals is not known exactly, which has led to development of new devices that will combine location-aware technology (e.g., GPS) with proximity loggers [65]. The temporal resolution of proximity logger data (often programmed to record continuously, [12]) will typically far exceed that of location data (e.g., from GPS), predominantly due to battery limitations. When proximity logger data are combined with GPS, proximity data can be incorporated into the calculation of the jPPA by computing the jPPA for only those times where the proximity logger identifies a contact to have occurred (i.e., τ in equation (6)). Such an extension will allow researchers to more precisely delineate regions where contacts occur when using the jPPA and incorporate spatially aware technology (e.g., GPS) to study the landscape context (habitat, topography, connectivity) associated with interactive behavior.

### Insights from the white-tailed deer examples

One of the most important outcomes of the development of new algorithms for studying inter-individual interaction is the application of these methods to discover previously undocumented biological insights of animal movement behavior. The results from the white-tailed deer example depict only three selected cases, selectively chosen to demonstrate different scenarios of interaction, and should not be used to make more general inferences about deer movement ecology. However, we can make some interesting conclusions about the specific individuals in our study to provide context for future analysis. Specifically, dyad 1 consisted of two adult males that were monitored concurrently over an approximate 2-week period, which fell within the breeding season of deer in this study area [66]. The results from our jPPA analysis for Dyad 1 suggest that any interaction may be related to brief and sporadic encounters. Biologically, this scenario is realistic considering that male deer do not socialize during the breeding season, but may combat with each other only temporarily, or may use similar areas when an estrous female is encountered. Dyad 2 consists of an adult male and adult female during the winter months suggesting that the interactive behavior observed in this dyad may be related to mating behavior (i.e., courting), which typically occurs on the Oklahoma, USA study area from 4 November to 24 December, with most breeding occurring from 18 November to 2 December [66]. In this case, jPPA analysis revealed that the potential courting behavior occurred in a specific wooded region of the study area (i.e., localized spatial extent), containing higher levels of percent canopy cover in comparison with other areas of the PPA ranges and OZ. The pair of males in dyad 3 are young adults that tend to form bachelor groups during spring and summer [67]. With dyad 3, the concurrent tracking period occurred from 15 February to 21 March, and the large jPPA area throughout their individual movement ranges may be an indication of the initiation of a bachelor pair (bachelor groups could be identified when analyzing *k* > 2 individuals). Habitat analysis revealed very little difference between the percent canopy cover within the PPA ranges, the OZ, and the jPPA, which would be expected considering the strong level of inter-individual interaction observed, and the general habitat needs of male deer following the breeding season, and during the antler shedding and growing cycle.

In general, it was also found that percent canopy cover estimates obtained using the telemetry points, differed from those from polygon-based home ranges. The general trend observed was that mean percent canopy cover was greater when the estimates were obtained from the telemetry points. One insight from this is that deer may be preferentially using forest edges [68], which when PPA home ranges are computed, the home range will include some open areas nearby as well. Then, it is interesting that the jPPA percent canopy cover values from dyad 2 were associated with higher percent canopy cover values than from the telemetry points, suggesting that this interaction phase occurred within a core forest area, as opposed to edge habitat. The problem of obtaining different results from habitat analysis when using telemetry points as the spatial unit vs. home range polygons as the spatial unit is well documented [69] and remains an ongoing challenge to the study of habitat composition from wildlife tracking data.

We hypothesize that using the jPPA to examine habitat use can reveal social behaviour and interactions of animals that may be driven by underlying landscape features (e.g., use of a shared or rare resource such as when animals visit watering holes, which could result in greater contact and interaction) or vice versa (use of landscape features is driven by social behaviour or interactions; e.g., during mating, animals may seek certain habitat types). Specifically, in dyad 3 from this study, we identified how the use of dense canopy cover within the jPPA would indicate generally important resources to male deer during winter (i.e., post-rut), which require greater energy and nutrition to recover from the stresses of the rut and for development of antlers in the spring [70, 71]. Knowing where and when habitat types and resources are used can facilitate management targeted at social groups (e.g., herds), rare resources where animals may be in contact (e.g., water sources), or during important life history phases (e.g., during mating).

### Further applications of jPPA analysis

We envision that the jPPA approach will be attractive to many wildlife ecologists because comparing jPPA polygons with ancillary geographic datasets is straightforward within a geographic information system (GIS) and accommodates similar spatial analyses as commonly applied to wildlife home ranges. The jPPA offers a new approach aimed specifically at *mapping* areas across the landscape where inter-individual interaction occurs. Wildlife researchers can use the jPPA polygons to test spatial hypotheses related to how interactive movement behaviour relates to underlying environmental variables. For example, researchers can estimate habitat composition associated with interactive behaviour using jPPA polygons in a similar manner to how composition is analyzed within home ranges, core areas, and utilization distributions [69]. We demonstrate such a process using a simple example where a single variable (percent canopy cover) is mapped across our study area. However, there is clear potential to use jPPA alongside multiple mapped covariates (e.g., landcover, topography, or weather data) to develop more sophisticated spatial analyses of resource selection to uncover greater biological insight into interactive behaviour. When performing habitat analysis from tracking data, it is important to consider how the organism perceives the environment, relating not only to the composition of habitat but also spatial pattern and scale [72–74]. As an alternative to pixel-based raster data, landscape features can be represented as distinct habitat ‘patches’, using a polygon data format, linear features, like roads, represented as lines, and other features, such as oil and gas well-sites, represented as points. In many species it will be interesting to examine how interactive behaviour is associated with these other types of features on the landscape (e.g., [19]). Output from jPPA analysis can be easily integrated with other geographic datasets within a GIS allowing more sophisticated spatial analysis of interactive movement behaviour.

The spatial patterns associated with the jPPA areas can also be used to understand biological processes associated with different interactive behaviours. For example, the configurational properties of jPPA regions may provide important information on the type of social interaction occurring. When jPPA regions are small and patchy relative to the home range and OZ it may be evidence of random encounters occurring across the landscape. Conversely, when the jPPA region is small but contiguous, the interaction observed may be an indication spatially localized resources being used simultaneously. When the jPPA covers a large proportion of the overlap zone, it is evidence of a sustained high-level of interactive behaviour easily corroborated by one of the many interaction statistics currently available [25, 26]; as evidenced by deer dyad 3 in our analysis. Further, more detailed analysis offers the potential to quantify which properties of the jPPA are associated with different social behaviour patterns. Polygon-shape indices, widely applied in the study of landscape patterns [75], offer potentially valuable metrics which could be included into the analysis of jPPA polygon regions in order to quantify, for example, compact or patchy shapes.

## Conclusion

The study of inter-individual interactions in wildlife is important to many population-level processes and is thus of special interest to wildlife managers. Here we have demonstrated a new approach for mapping areas of inter-individual interaction from wildlife tracking data. The new approach extends a previously developed home range estimator [34] in-order to delineate areas of joint accessibility (termed the jPPA) between two (or more) simultaneously tracked animals. Maps of the jPPA provide researchers with a new spatial unit from which habitat analysis can be easily conducted and directly related to inter-individual interactions. We also demonstrate the application of the jPPA approach in habitat analysis by exploring the percent canopy cover with three deer dyads tracked using GPS tracking data. Spatial patterns of the jPPA, especially in relation to the OZ and home range areas, can be useful indication of the type of inter-individual interaction occurring in a dyad (e.g., random encounters vs. joint spatial use of local resources). Extending current indices that test for the presence or absence of interaction behaviour and new developments capable of mapping inter-individual interactions in space and time (such as the jPPA) are essential for studying the complex and infrequent social encounters of wildlife using remote tracking data. Finally, in order to assist other researchers wishing to utilize the jPPA in their own research, we make openly available code for computing the jPPA in the statistical software R [76] (for access to R code and tools please see: http://jedalong.github.io/wildlifeTG/).

## Additional file

Additional file 1:
**Derivation of biased correlated random walks (BCRW) as implemented in the simulation study.** (ZIP 26.5 kb)
