# Supplementary material for: Mapping areas of spatial-temporal overlap from wildlife tracking data
Source: Mov Ecol. 2015 Nov 1;3:38. doi: 10.1186/s40462-015-0064-3 (PMC4628783; doi:10.1186/s40462-015-0064-3)
Supplement: Additional file 1: — Derivation of biased correlated random walks (BCRW) as implemented in the simulation study. (ZIP 26.5 kb) [file 40462_2015_64_MOESM1_ESM.zip › SuppMat-A - 29-06-2015.docx]

**Supplementary Material A:**

**Derivation of biased correlated random walks (BCRW) as implemented in the simulation study.**

A correlated random walk (CRW) takes the form [1]:

$x_{t+1}=d_{t}\cos\left( \theta_{t} \right)+x_{t}$ (a1)

$y_{t+1}=d_{t}\sin\left( \theta_{t} \right)+y_{t}$ (a2)

Where *x_t_* and *y_t_* are the spatial coordinates of the CRW at time *t*, *d_t_* is the step-length at time *t* and *θ_t_* is the step direction at time *t*. Typically, to simulate a CRW, *d* is taken as a random draw from a step-length distribution; here, we model *d* as a random draw from a *χ* distribution multiplied by a step-length scaling parameter *h*. The correlation effect is initiated via the direction parameter *θ*, typically modelled as:

$\theta_{t}=\theta_{t-1}+\vartheta_{t}$ (a3)

Where *ϑ_t_* is the change in direction from *θ_t-1_* to *θ_t_*. In practice, simulating this effect is achieved through selecting *θ_t_* as a random draw from a wrapped circular distribution centered on *θ_t-1_* with a parameter *r*, which controls the concentration of the wrapped distribution about this mean, and thus the correlation effect. Here, we use the wrapped normal distribution as the circular distribution, but the wrapped Cauchy distribution is also commonly employed.

A biased correlated random walk (BCRW) can then be modelled by further adjustments to the calculation of the correlation effect on *θ_t_* in eq. a3. Following Barton *et al.* [2], a BCRW can be defined by weighting the directional correlation (persistence) and bias effects as:

$\theta_{t}=\left( 1-\beta_{t} \right)\theta_{t-1}+\beta_{t}\varphi_{t}$ (a4)

Where again *θ_t_* is the movement direction at time *t*, *φ_t_* is the bias direction (see below) and *β_t_* is used to differentially weight between correlated (persistence) and biased movement. Note for *β_t_* = 0 it is an ordinary CRW, and for *β_t_* = 1, it is a biased random walk. The parameter *β_t_* then represents the magnitude of the bias effect, and is typically modelled by considering the current distance (*δ_t_*) between the individual and the object to which the bias occurs:

$\beta_{t}=\tanh\left( b\delta_{t}^{c} \right)$ (a5)

Where *δ_t_* is the current distance between the individual and the object to which the bias occurs, and *b* and *c* are parameters that control the strength and distance decay of the bias effect. The parameter *b*, regarded as the strength parameter, is typically given a value of *b* > 0. The parameter *c* is the bias distance decay effect, where *c* < 0 reflects decreasing bias with distance between the individual and the bias object, and *c* > 0 reflects increasing bias with distance between the individual and the bias object. Here, we simulate the jointly correlated and biased effect on *θ_t_* by taking a random draw from a wrapped normal distribution, centered on the value computed in (eq. a4) and using a concentration parameter (*ρ*) similar to parameter *r* from the ordinary CRW [3].

The BCRW framework, as outlined above, is used to simulate three different movement scenarios where inter-individual interaction would be expected between two individuals, A and B (see Figure 3 in the text).

1. Grouping
2. Leading/Following
3. Joint resource use

In scenario i) grouping, the bias effect occurs in both individuals, and is from their location to the location of a third independent CRW [4], which models the location (i.e., movement) of a group/herd center. In scenario ii) leading/following, the bias effect is modelled as occurring from the second individual towards the current location of the first [5]. That is, the bias effect occurs only in individual B, and is directed from $\left( x_{t-1}^{B},y_{t-1}^{B} \right)$ to $\left( x_{t}^{A},y_{t}^{A} \right)$. Finally, in scenario iii) joint resource use, the bias effect is towards the center of a joint resource patch (similar to [2]).

In reality, animals switch between phases of interactive and non-interactive behavior. To emulate the switching between interactive and non-interactive behavior, we implemented a phase switching effect [6] into the simulations, whereby animals switch from biased to unbiased phases with probability *p_a_* and from unbiased to biased phases with probability *p_b_*. In scenarios i) and ii) when an animal is in an unbiased phase, it continues movement with an ordinary CRW via eq. a1-3. In scenario iii) the animals A and B are given disjoint home range centers on either side of the joint resource patch. When in an unbiased phase (i.e., no interaction with the joint resource patch), the animals move with a BCRW towards their home range center, to simulate typical home range behavior. In all scenarios, the animals begin in the unbiased phase.

The simulation procedure as outlined above requires the selection of a number of parameters as defined in Table A1 below. We vary these randomly across the range of values indicated in Table A1 in order to explore potentially different interaction levels for each of the three scenarios. The R code used to simulate each of these scenarios is also provided alongside this supplementary material.

Table A1: How parameters used to formulate the biased correlated random walk (BCRW) were chosen in the simulated study. Except for the integer number of steps, parameters were randomly chosen from a uniform distribution truncated by the parameter range.

| **Parameter** | **Description** | **Implemented in Simulations** |
| --- | --- | --- |
| *n* | Number of steps in the random walk (i.e., number of telemetry fixes). | integer; 500 < *n* < 2000 |
| *h* | CRW step-length parameter, scaling factor for random draw from *χ* distribution. | 1 < *h* < 5 |
| *r* | CRW correlation concentration parameter, using a wrapped Normal distribution. | 0 < *r* < 0.5 |
| *ρ* | BCRW correlation concentration parameter, using a wrapped Normal distribution. | 0.8 < *ρ* < 0.9 |
| *b* | BCRW bias strength parameter, typically *b* > 0. | 1 < *b* < 2 |
| *c* | BCRW bias distance decay parameter; *c* < 0 – bias decreases with separation distance; *c* > 0 – bias increases with separation distance. | -0.3 < *c* < 0.3 |
| *p_a_* | Transition probability from biased to unbiased phase. | 0.001 < *p_a_* < 0.05 |
| *p_b_* | Transition probability from unbiased to biased phase. | 0.001 < *p_b_* < 0.05 |

**References**

1. Turchin P: *Quantitative Analysis of Movement: Measuring and Modeling Population Redistribution in Animals and Plants*. Sinauer Associates; 1998.

2. Barton K a., Phillips BL, Morales JM, Travis JMJ: **The evolution of an “intelligent” dispersal strategy: biased, correlated random walks in patchy landscapes**. *Oikos* 2009, **118**:309–319.

3. Fronhofer EA, Hovestadt T, Poethke H-J: **From random walks to informed movement**. *Oikos* 2013, **122**:857–866.

4. Langrock R, Hopcraft JGC, Blackwell PG, Goodall V, King R, Niu M, Patterson T a., Pedersen MW, Skarin A, Schick RS: **Modelling group dynamic animal movement**. *Methods Ecol Evol* 2014, **5**:190–199.

5. Long JA, Nelson TA, Webb SL, Gee KL: **A critical examination of indices of dynamic interaction for wildlife telemetry studies.** *J Anim Ecol* 2014, **83**:1216–1233.

6. Morales J, Haydon D, Frair J, Holsinger KE, Fryxell JM: **Extracting more out of relocation data: building movement models as mixtures of random walks**. *Ecology* 2004, **85**:2436–2445.
